# Effectiveness of the electronic Community Health Information System on maternal continuum of care completion in rural East Shewa Zone, Eastern Ethiopia: A prospective controlled quasi-experimental study

**DOI:** 10.1371/journal.pdig.0001775

**Published:** 2026-10-07

**Authors:** Serbesa Dereje Degaga, Solomon Shiferaw, Girma Taye Aweke

**Affiliations:** School of Public Health, College of Health Sciences, Addis Ababa University, Addis Ababa, Ethiopia; Yonsei University College of Medicine, KOREA, REPUBLIC OF

## Abstract

Maternal mortality in Ethiopia remains high, and strengthening the continuum of maternal care is essential for reducing preventable deaths. The electronic Community Health Information System was introduced to improve service delivery through decision support, client tracking, and electronic referrals, but evidence of its effect in routine settings is limited. This study assessed the effect of the eCHIS’s Reproductive, Maternal, Newborn, Child Health and Nutrition module intervention on completion of the maternal continuum of care. A prospective controlled pre–post quasi-experimental study design was conducted in Primary Health Care Units. Baseline data were collected from January 5 to February 5, 2025, enrolling pregnant women with twenty six weeks of gestation or earlier. Participants were followed for nine months, and end-line data collected that year. Maternal care outcomes came from antenatal, delivery, and postnatal registers, with verification interviews when records were incomplete. The primary outcome was completion of the maternal continuum of care. Intervention effects were estimated using a Difference in Differences fixed effects model with cluster robust standard errors. Follow-up was completed for 409 women in each group (97.5%). Baseline continuum of care completion was comparable across groups. By endline, completion increased from 19.6% to 47.7% in the intervention group versus 19.1% to 23.7% in controls. The eCHIS-RMNCHN intervention produced a 23.5% point improvement in full continuum of care completion (β = 0.235; 95% CI: [0.155–0.314]), with significant gains in ANC4+ and ANC4 + combined with SBA. Implementation of the eCHIS-RMNCHN module intervention was associated with substantial improvements in maternal continuum-of-care completion and key intermediate indicators. Strategies guided by the CFIR ERIC framework helped address local barriers to digital system use. Sustained implementation, leadership engagement, technical support, community involvement, and continuous performance monitoring are essential for maintaining these improvements and informing scale up efforts.

## Author summary

Maternal health services are most effective when women receive continuous care during pregnancy, childbirth, and the early postnatal period. However, many women in Ethiopia do not complete this continuum of care. This study evaluated whether the electronic Community Health Information System Reproductive, Maternal, Newborn, Child Health and Nutrition module could improve continuity of maternal care in routine primary healthcare settings.

We compared women receiving care in primary healthcare units implementing the eCHIS-RMNCHN module with women receiving conventional services. Participants were followed from early pregnancy through childbirth and the postnatal period. Core functions of the eCHIS-RMNCHN module included digital client tracking, appointment reminders, clinical decision support, electronic referral, and structured counseling. These functions were supported by implementation strategies such as community follow-up, leadership engagement, technical assistance, and routine performance monitoring.

Using a Difference-in-Differences analysis, we found that the intervention was associated with a 19.3-percentage-point greater improvement in completion of four or more antenatal care visits, a 17.4-percentage-point greater improvement in four or more antenatal visits combined with skilled birth attendance, and a 23.5-percentage-point greater improvement in full maternal continuum-of-care completion. Sensitivity and wild cluster bootstrap analyses generally supported these findings.

These results suggest that the eCHIS-RMNCHN module can strengthen continuity of maternal care when its core functions are supported by effective implementation strategies.

## Introduction

Maternal mortality remains a critical global health concern. In 2023, an estimated 260,000 women died from causes related to pregnancy and childbirth which implies one death every two minutes [1,2]. Most of these deaths are preventable and sub-Saharan Africa contributes nearly 70 percent of the global burden [2,3].

Ethiopia has achieved substantial progress in reducing maternal mortality over the past two decades. The maternal mortality ratio declined from 953 per 100,000 live births in 2000–267 in 2020, surpassing the average regional pace of reduction [4,5]. Despite these achievements, the current level remains almost four times higher than the Sustainable Development Goal target. Country-level assessments supported by independent research have shown that preventable factors driving maternal mortality include inadequate antenatal care coverage, limited skilled birth attendance, delays in accessing emergency obstetric interventions, and marked inequities between urban and rural settings [6,7].

Strengthening the continuum of maternal health care is widely recommended as an approach to reduce preventable maternal morbidity and mortality [8]. Mobile health technologies have been shown to enhance antenatal care adherence, skilled birth attendance, and postnatal care use in low and middle income countries [9]. In a bid to augment the quality of services and enhance the continuum of care (COC) at the grassroots, the Ministry of Health launched the electronic Community Health Information System (eCHIS) in 2018 as a part of its mHealth initiative & high-priority Information revolution agenda [10].

eCHIS is designed to digitize Ethiopia’s Health Extension Program (HEP) to advance universal access to primary health care at the community level. It has four core modules: Digital Family Folder; Reproductive, Maternal, Newborn, Child Health and Nutrition; Disease Prevention and Control; and Logistics, Supply and Management [11,12]. Building on this platform, the eCHIS-RMNCHN module enables comprehensive maternal assessment and evidence-based decisions, standardizes counseling on obstetric danger signs across pregnancy, childbirth, and postpartum, maintains continuity of care through longitudinal client tracking with integrated e-referrals, and supports feedback from health-center midwives on referrals initiated by Health Extension Workers [13].

Although emerging evidence from Ethiopia suggests that the electronic Community Health Information System (eCHIS) improves maternal health services and strengthens the continuum of care, the empirical base is limited [14]. To date, merely one study has specifically examined eCHIS effects on the maternal continuum of care. It employed a single-group pre–post design without a concurrent comparison group. Such a design cannot disentangle program effects from secular trends, seasonality, or co-occurring interventions, thereby limiting causal inference. Consequently, robust evidence to guide implementation and inform scale-up remains insufficient.

The present study aimed to assess the effect of eCHIS-RMNCHN module intervention on completion of the maternal continuum of care using a prospective controlled before and after design in rural East Shewa zone, Easten Ethiopia.

## Results

### Socio-demographic and reproductive characteristics

A total of 839 pregnant women were enrolled at baseline, including 418 in the intervention arm and 421 in the control arm. At the nine-month endline assessment, outcome data were successfully obtained for 409 women in each group, resulting in an overall follow-up rate of 97.5%. Attrition was low and nearly identical across arms: 12 women (2.85%) were lost to follow-up in the control group and 9 women (2.15%) in the intervention group.

At baseline, the age distribution differed between the two groups, with a higher proportion of women aged 25–34 years in the intervention arm and a higher proportion of women aged 15–24 years in the control arm (p < 0.001). Educational status was generally similar across groups, although primary education was slightly more common in the control arm. Orthodox Christianity was the predominant religion in both groups, but it was more frequent in the control arm, whereas Protestant and Catholic affiliations were comparatively higher in the intervention arm (p < 0.001). Most women in both groups belonged to the Oromo ethnic group, although a higher proportion of Amhara women was observed in the intervention arm (p = 0.002). Nearly all participants in both groups were married.

With respect to reproductive history, significant differences were observed in parity, with primiparity being more common in the intervention group and multiparity being more common in the control group (p = 0.001). Most pregnancies were planned in both arms; however, unplanned pregnancies were slightly more frequent among women in the intervention group (p = 0.053). In addition, a history of poor fetal outcome was more commonly reported among women in the intervention group compared with the control group (14.2% vs. 8.1%, p = 0.008). As the same cohort of women was followed from baseline to endline, these socio-demographic and reproductive characteristics remained unchanged over time Table 1.

**Table 1:** Socio-demographic and reproductive characteristics of participants by study arm at baseline and endline.

Variable	Category	Baseline (n = 839)		p-value	Endline(n = 818)		p-value
		Control(n = 421)	Intervention (n = 418)		Control (n = 409)	Intervention(n = 409)	
		Baseline Control n (%)	Baseline Intervention n (%)		Endline Control n (%)	Endline Intervention n (%)	
**Age group (years)**	15–24	189 (44.9)	94 (22.5)	<0.001	184 (45.0)	92 (22.5)	<0.001
	25–34	206 (48.9)	284 (67.9)		200 (48.9)	278 (68.0)	
	35–49	26 (6.2)	40 (9.6)		25 (6.1)	39 (9.5)	
**Women’s education**	Cannot read/write	63 (15.0)	65 (15.6)	0.112	61 (14.9)	64 (15.6)	0.121
	Can read/write	124 (29.5)	147 (35.2)		121 (29.6)	144 (35.2)	
	Primary education	197 (46.8)	162 (38.8)		191 (46.7)	158 (38.6)	
	Secondary and above	37 (8.8)	44 (10.5)		36 (8.8)	43 (10.5)	
**Religion**	Orthodox	345 (81.9)	302 (72.2)	<0.001	335 (81.9)	295 (72.1)	<0.001
	Protestant	67 (15.9)	92 (22.0)		65 (15.9)	90 (22.0)	
	Catholic	0 (0.0)	19 (4.5)		0 (0.0)	19 (4.6)	
	Muslim	8 (1.9)	3 (0.7)		8 (2.0)	3 (0.7)	
	Wakefata	1 (0.2)	2 (0.5)		1 (0.2)	2 (0.5)	
**Ethnicity**	Oromo	395 (93.8)	363 (86.8)	0.002	384 (93.9)	355 (86.8)	0.002
	Amhara	16 (3.8)	39 (9.3)		15 (3.7)	38 (9.3)	
	Others	10 (2.4)	16 (3.8)		10 (2.4)	16 (3.9)	
**Marital status**	Married	417 (99.0)	410 (98.1)	0.240	405 (99.0)	401 (98.0)	0.245
	Others	4 (1.0)	8 (1.9)		4 (1.0)	8 (2.0)	
**Parity**	Primipara	117 (27.8)	166 (39.7)	0.001	114 (27.9)	163 (39.9)	0.001
	Multipara	267 (63.4)	217 (51.9)		259 (63.3)	212 (51.8)	
	Grand multipara	37 (8.8)	35 (8.4)		36 (8.8)	34 (8.3)	
**Planned pregnancy**	Yes	359 (85.3)	334 (79.9)	0.040	349 (85.3)	327 (80.0)	0.042
	No	62 (14.7)	84 (20.1)		60 (14.7)	82 (20.0)	
**History of poor fetal outcome**	Yes	34 (8.1)	59 (14.1)	0.005	33 (8.1)	58 (14.2)	0.005
	No	387 (91.9)	359 (85.9)		376 (91.9)	351 (85.8)	
**Family wealth index**	Low	299 (71.0)	191 (45.7)	<0.001	290 (70.9)	187 (45.7)	<0.001
	Medium	48 (11.4)	92 (22.0)		47 (11.5)	90 (22.0)	
	Higher	74 (17.6)	135 (32.3)		72 (17.6)	132 (32.3)	
**Age at first marriage**	Less than 18 years	59 (14.0)	101 (24.2)	<0.001	57 (13.9)	99 (24.2)	<0.001
	18 years and above	362 (86.0)	317 (75.8)		352 (86.1)	310 (75.8)	
**Time of booking for ANC**	After 12 weeks	373 (88.6)	360 (86.1)	0.281	361 (88.3)	352 (86.1)	0.347
	Within 12 weeks	48 (11.4)	58 (13.9)		48 (11.7)	57 (13.9)	
**Content of ANC**	Inadequate	142 (33.7)	170 (40.7)	0.038	118 (28.9)	81 (19.8)	0.003
	Adequate	279 (66.3)	248 (59.3)		291 (71.1)	328 (80.2)	
**Knowledge of at least two danger signs**	No	180 (42.8)	164 (39.2)	0.300	156 (38.1)	78 (19.1)	<0.001
	Yes	241 (57.2)	254 (60.8)		253 (61.9)	331 (80.9)	
**Distance to health facility**	More than 1 hour	143 (34.0)	189 (45.2)	<0.001	139 (34.0)	185 (45.2)	0.001
	≤1 hour	278 (66.0)	229 (54.8)		270 (66.0)	224 (54.8)	
**Health facility readiness level**	High readiness	73 (17.3)	65 (15.6)	0.690	71 (17.4)	64 (15.6)	0.698
	Low readiness	323 (76.7)	331 (79.2)		314 (76.8)	324 (79.2)	
	Medium readiness	25 (5.9)	22 (5.3)		24 (5.9)	21 (5.1)	

Historical pre-intervention maternal health service performance was broadly comparable between the intervention and control PHCUs in 2023 and 2024. No statistically significant between-group differences were observed in ANC1, ANC4, skilled birth attendance, PNC within 48 hours, or early PNC coverage in either year Table 2.

**Table 2:** Historical pre-intervention performance of maternal health service indicators in intervention and control PHCUs, 2023–2024.

Indicator	Intervention, %	Control, %	Difference in percentage points, intervention − control (95% CI)	p-value
	2023 / 2024	2023 / 2024	2023 / 2024	2023 / 2024
ANC1	63.8 / 68.7	66.3 / 71.6	−2.5 [−9.0 - 4.0] / −2.9 [−9.0 - 3.4]	0.448 / 0.370
ANC4	52.1 / 58.4	53.9 / 55.3	−1.8 [−8.6 - 5.0] / 3.1 [−3.7 to 9.7]	0.601 / 0.376
Skilled birth attendance	56.6 / 57.9	52.3 / 54.6	4.3 [−2.4 - 11.0] / 3.3 [−3.4 - 10.0]	0.211 / 0.341
PNC within 48 hours	50.9 / 58.8	55.8 / 64.1	−4.9 [−11.6 -1.8] / −5.3 [−11.9 - 1.3]	0.155 / 0.116
Early PNC	59.8 / 65.7	58.3 / 67.2	1.5 [−5.2 - 8.2] / −1.5 [−7.9 - 4.9]	0.635 / 0.645

### Changes in maternal health care continuum indicators between baseline and end line

At baseline, the levels of maternal continuum of care indicators were comparable between the intervention and control groups. In the control group, the proportion of women who completed four or more antenatal care visits was 42.1%, while 31.1% achieved both ANC4 and skilled birth attendance, and 50.9% received postnatal care within 48 hours. The overall completion of the maternal continuum of care in the control group was 19.1% at baseline and increased slightly to 23.7% at endline.

In the intervention group, 37.9% of women achieved four or more antenatal care visits at baseline, which increased substantially to 61.9% at endline. Similarly, the proportion attaining both ANC4 and skilled birth attendance increased from 25.7% to 48.2%, and postnatal care coverage increased from 51.1% to 63.1% by the endline assessment. Notably, the proportion of women completing the full continuum of maternal care increased from 19.6% at baseline to 47.7% at endline in the intervention group, representing a marked improvement compared to the control group. Changes in maternal continuum-of-care indicators between baseline and endline are presented in Fig 1.

**Fig 1:**
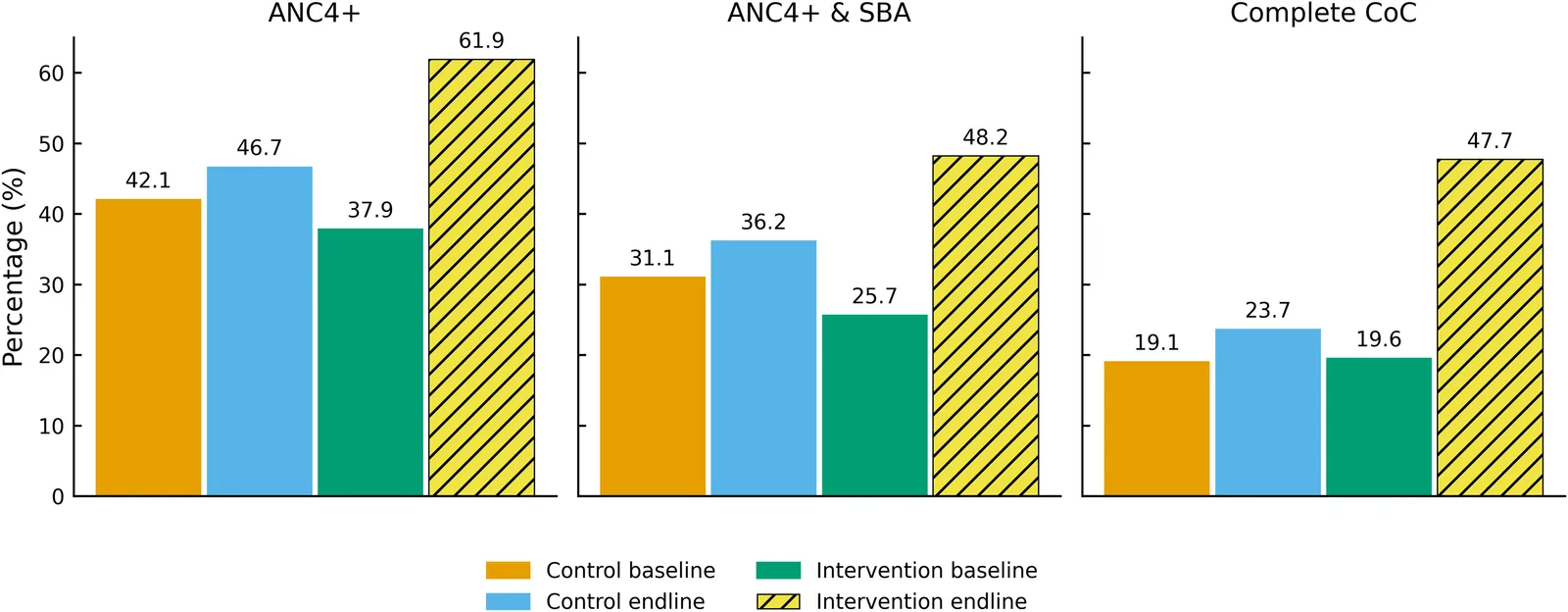
Changes in maternal health care continuum indicators between baseline and endline in the intervention and control groups, East Shewa Zone, 2025.

### Effect of the eCHIS-RMNCHN intervention on maternal continuum of care

Fig 2 illustrates the impact of the eCHIS-RMNCHN intervention on key maternal continuum of care indicators. At baseline, completion of the full continuum of care (ANC4+ with skilled birth attendance and postnatal care within 48 hours) was comparable in intervention and control facilities (19.6% vs 19.1%). By endline, completion increased modestly in the control group (to 23.7%) but rose substantially in the intervention group (to 47.7%). The Difference-in-Differences fixed-effects model showed that the intervention was associated with a **23.5 percentage-point** absolute increase in complete continuum of care completion (β = 0.235, 95% CI: [0.155–0.314]; p < 0.001), after accounting for woman-level fixed effects and clustering at facility level.

**Fig 2:**
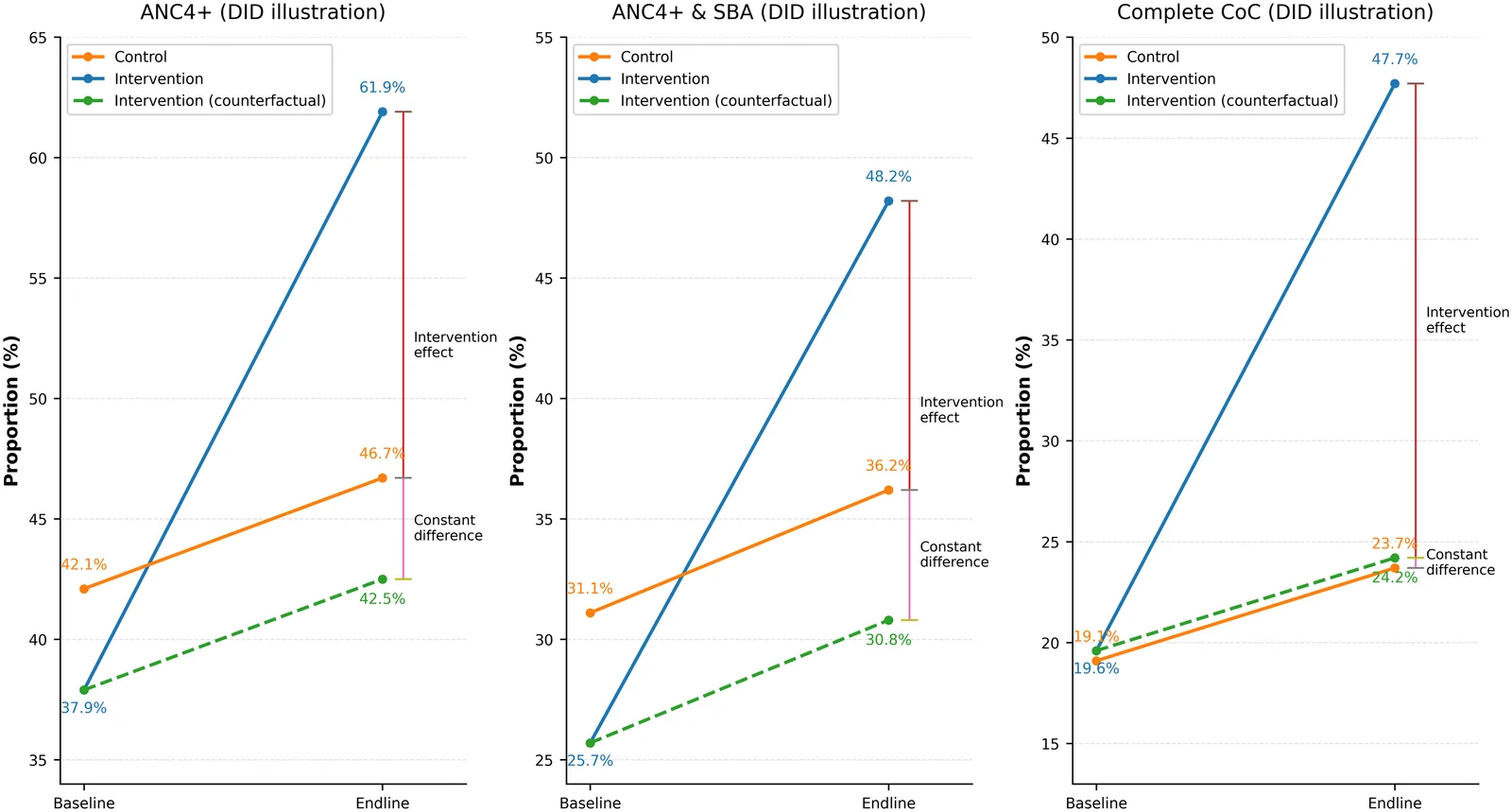
DID illustration of maternal health care continuum indicators in the intervention and control groups, East Shewa Zone, 2025.

For ANC4 + coverage, baseline levels were 37.9% in the intervention arm and 42.1% in the control arm. At endline, ANC4 + increased to 61.9% in the intervention arm compared with 46.7% in the control arm The DiD estimate indicated a 19.3-percentage-point greater improvement associated with the eCHIS-RMNCHN intervention (β = 0.193, 95% CI: [0.050–0.337]; p = 0.014).

Similarly, the proportion of women achieving both ANC4+ and skilled birth attendance (ANC4 + & SBA) increased from 25.7% to 48.2% in intervention facilities and from 31.1% to 36.2% in control facilities. The corresponding DiD coefficient was **17.4 percentage points** (β = 0.174, 95% CI: [0.037–0.310]; p = 0.018).

Overall, the pattern of results was consistent with a positive intervention-associated improvement in intermediate and complete continuum-of-care outcomes following implementation of the integrated eCHIS-RMNCHN intervention. The Difference-in-Differences estimates for the maternal continuum-of-care outcomes are presented in Fig 2.

Given the limited number of PHCU clusters, wild cluster bootstrap-t inference was conducted as a small-sample robustness analysis. The intervention effect remained statistically significant for ANC4+ (bootstrap P = 0.024; 95% CI [0.023–0.35], combined ANC4+ and skilled birth attendance (bootstrap p = 0.006; 95% CI [0.028–0.336], and complete continuum-of-care completion (bootstrap p < 0.001; 95% CI: [0.150–0.324] Table 3.

**Table 3:** Primary Difference-in-Differences estimates and wild cluster bootstrap robustness analysis for maternal continuum-of-care outcomes.

Outcome	Primary DiD estimate	Cluster-robust p-value	Wild bootstrap 95% confidence set	Wild bootstrap p-value
ANC4+	0.193	0.014	[0.023–0.359]	0.024
ANC4+ and SBA	0.174	0.018	[0.028–0.336]	0.006
Complete CoC	0.235	<0.001	[0.150–0.324]	<0.001

Sensitivity analyses were conducted to assess the robustness of the intervention-effect estimates to observed baseline imbalances. The adjusted estimates were generally similar in magnitude to the primary estimates. The intervention effect remained statistically significant for ANC4+ and complete maternal continuum of care. For ANC4+ with skilled birth attendance, the adjusted estimate remained positive but did not reach statistical significance Table 4.

**Table 4:** Primary and sensitivity-adjusted Difference-in-Differences estimates for maternal continuum-of-care outcomes.

Outcome	Primary DiD estimate (95% CI)	p-value	Sensitivity-adjusted DiD estimate (95% CI)	p-value
ANC4+	0.193 [0.050–0.337]	0.014	0.169 [0.006–0.333]	0.044
ANC4+ and SBA	0.174 [0.037–0.310]	0.018	0.153 [−0.013–0.319]	0.067
Complete CoC	0.235 [0.155–0.314]	<0.001	0.242 [0.138–0.347]	<0.001

## Discussion

This study revealed that implementation of the eCHIS RMNCHN module resulted in substantial improvements in the maternal continuum of care. Completion increased from 19.6% at baseline to 47.7% at endline in the intervention arm, compared with a more modest increase from 19.1% to 23.7% in the comparison arm. The corresponding Difference-in-Differences estimate indicated a 23.5-percentage-point greater improvement associated with implementation of the eCHIS-RMNCHN intervention. These findings provide evidence that an integrated digital health systems and implementation package can strengthen continuity of maternal care under routine primary healthcare conditions.

The intervention also strengthened intermediate indicators. Coverage of ANC4 plus increased from 37.9 percent to 61.9 percent in the intervention arm and from 42.1 percent to 46.7 percent in the control arm. The Difference in Differences estimate showed a 19.3 percentage point improvement. Similarly, the combined indicator of ANC4 plus and skilled birth attendance increased from 25.7 percent to 48.2 percent in the intervention arm and from 31.1 percent to 36.2 percent in the control arm. The corresponding Difference in Differences coefficient showed a 17.4 percentage point improvement. These findings indicate that the digital system supported progression through multiple components of the maternity care pathway.

The observed improvement may have resulted from the combined functions of the intervention, including longitudinal client tracking, appointment reminders, electronic referral, structured counseling, clinical decision support, follow-up of missed visits, community engagement, technical mentorship, leadership involvement, and routine performance monitoring. Together, these mechanisms may have supported women’s continuity through antenatal care, skilled birth attendance, and early postnatal care.

The implementation strategies applied in this study were informed by a prior assessment of eCHIS implementation determinants conducted in the same setting using the CFIR ERIC framework. Because these strategies were tailored to locally identified barriers such as limited leadership engagement, gaps in technical support, weak community follow up, and inconsistent performance monitoring, they likely contributed to the substantial improvements observed in ANC4 plus, skilled birth attendance, and completion of the full continuum of care.

Recent national evidence reported that intervention-adjusted ANC4 + coverage and process-quality-adjusted skilled birth attendance coverage were 16% and 19% [15] respectively, highlighting substantial gaps between service contact and effective coverage. These estimates are not directly comparable with the 47.7% observed in our study because our outcome was a contact-based composite of ANC4 + , skilled birth attendance, and postnatal care within 48 hours and was not adjusted for receipt of recommended clinical interventions, adherence to clinical standards, or process quality. Nevertheless, the findings emphasize that improved continuity of contact does not necessarily ensure high-quality care. Future eCHIS evaluations should therefore incorporate measures of service content, process quality, and patient outcomes.

The improvement in ANC4 plus observed in this study was 19.3%, which is greater than improvements reported in several comparable digital health interventions. A randomized trial in Zanzibar demonstrated increased antenatal care attendance following a mobile phone intervention, although the magnitude of improvement was smaller than the gains found here [16]. Evidence from Ethiopia also shows that digital decision support and community health worker tools can strengthen maternal service utilization, but the extent of improvement varies depending on context, supervision, and system readiness [17].

Findings from other digital intervention studies conducted in Ethiopia further support this pattern. A locally developed mHealth platform evaluated in Semen Shewa showed significant increases in institutional delivery and postnatal care through appointment reminders and danger-sign messaging, although improvements in repeat ANC attendance were more modest [18]. Similarly, the ReMiND mobile application in India improved recognition of danger signs and some ANC processes, but gains in institutional delivery were not statistically significant [19]. These findings suggest that the combination of digital reminders alone may not be sufficient and that additional implementation strategies such as enhanced supervision, strengthened community linkages, and provider-focused mentorship may be necessary to achieve higher levels of ANC completion.

The present study incorporated strong leadership engagement, continuous mentorship, and regular refresher training. These elements likely facilitated prioritization of electronic Community Health Information System tasks. Enhanced community involvement and structured follow up of missed visits may also have contributed to earlier identification of pregnant women and improved adherence to scheduled visits. Together, these factors may have created more favorable conditions for improvement than those documented in previous digital interventions.

The improvement in ANC4 plus combined with skilled birth attendance, observed as a 17.4% increase, is comparable to findings from other mobile and digital platforms aimed at strengthening maternal care. Randomized and quasi-experimental studies have shown that digital tools supporting counseling, referral, and reminders typically yield improvements between 10% and 20% for key maternity indicators [19]. Evidence from Nigeria, India, and Ethiopia indicates that digital platforms frequently improve ANC attendance and counseling quality but have variable effects on skilled birth attendance [16,20,21]. More comprehensive digital health interventions targeting health facility data use and provider performance may achieve stronger gains. For example, a national HIS intervention package in Ethiopia demonstrated significant improvements in ANC4 + , SBA, and PNC by strengthening data quality, performance monitoring, and facility-level decision-making [14].

Findings from the recent eCHIS impact study in Ethiopia also align with the present study. The eCHIS platform significantly improved continuum of care completion by enhancing referral tracking, data quality, and follow up of pregnant women. The similarities between that trial and the current study suggest that digital community health systems are particularly effective when combined with structured supervision, performance review, and strong community-facility linkages.

Some studies, however, have reported more modest improvements. Across sub-Saharan Africa, assessments of antenatal and intrapartum care highlight persistent constraints in facility readiness, including shortages of skilled health professionals, limited infrastructure, and inconsistent availability of essential maternity services [22–24]. Such limitations can reduce the potential impact of digital health interventions on care completion. Additional barriers, such as transportation challenges, long distances to facilities, and financial constraints, further restrict women’s ability to translate digital reminders into completed service contacts [23]. Limited community participation and insufficient follow up mechanisms also contribute to smaller gains in some settings [24]. In contrast, the current intervention incorporated leadership engagement, regular mentorship, ongoing feedback, community involvement, and recognition of high-performing health workers. These elements likely reinforced motivation, improved data accuracy, and strengthened adherence to follow up protocols, resulting in the larger improvements observed.

These findings have important implications for maternal health programming in Ethiopia. Digital systems can substantially improve continuity of maternal care in contexts where completion of the care pathway remains low. The improvements observed in this study suggest that well-implemented digital interventions can contribute to national goals for maternal and newborn health and support progress toward the Sustainable Development Goal for reduction of maternal mortality. Integrating the electronic Community Health Information System into routine workflows will help sustain these gains. Continued investment in supportive supervision, capacity building, and regular performance review is essential, and improved interoperability with District Health Information Software 2 can further enhance efficiency and reduce duplication of reporting.

Overall, the study provides strong evidence that electronic community health information systems can improve maternal service uptake across the continuum of care. However, the aggregate intervention effect does not establish that the benefits of eCHIS were distributed equitably across all population groups. Future evaluations should therefore examine intervention effects across socio-demographic, socioeconomic, and other locally relevant characteristics. Such analyses are needed to determine whether digital health interventions reduce existing inequities or inadvertently reproduce them. Future research should also explore long-term sustainability, cost effectiveness, and opportunities for expansion to other regions.

### Strengths and limitations

This study has some important limitations. Primary health care units were not randomly assigned, which means unmeasured differences between facilities may still have influenced the results. Since only one study baseline measurement was available, the parallel trends assumption underlying the Difference-in-Differences analysis could not be tested empirically. Although baseline outcome levels were similar between the study arms, differential changes over time cannot be ruled out, as the woman-specific fixed-effects model controlled for time-invariant individual characteristics but not for unmeasured time-varying contextual or health-system factors. Sensitivity analyses addressing observed baseline imbalances produced estimates that were generally consistent with the primary findings; however, residual confounding remains possible.

Baseline and endline outcomes also referred to different pregnancy episodes and were not ascertained using fully identical procedures. Baseline outcomes referred to the most recent previous pregnancy and were collected retrospectively, with register cross-checking when records were available. Endline outcomes referred to the prospectively followed index pregnancy and were obtained mainly from facility registers, with verification of incomplete records. Although all participants had experienced a previous birth, recall error and differences in documentation completeness may have affected comparability between periods.

Differential outcome ascertainment cannot be excluded. Routine antenatal, delivery, and postnatal registers were the primary sources of endline outcomes, while missing or incomplete information was supplemented through verification interviews and active tracing. As eCHIS may improve both service delivery and documentation, part of the observed intervention-associated improvement may reflect increased service receipt, improved record completeness, or both.Despite these limitations, the study has important strengths. To our knowledge, this is the first study of its kind in Ethiopia to evaluate the effect of the eCHIS-RMNCHN module on completion of the maternal continuum of care. It was carried out under real-world conditions using a prospective controlled quasi-experimental design with a comparison group. The use of a Difference in Differences fixed-effects model, supported by cluster-robust standard errors and wild cluster bootstrap procedures, increased confidence in the findings. Following the same women over time and relying mainly on routine facility records reduced recall bias and allowed assessment of both intermediate outcomes and full completion of the continuum of care. In addition, the study generated clear and policy-relevant evidence, supported by a strong implementation-science perspective using the CFIR–ERIC framework to explain how and why the intervention produced the observed improvements.

## Conclusion

This study found that implementation of an integrated intervention comprising the eCHIS-RMNCHN module and CFIR–ERIC-informed implementation strategies was associated with substantial improvements in maternal continuum-of-care completion in rural East Shewa zone, Eastern Ethiopia. Significant gains were observed in ANC4 plus, ANC4 plus with skilled birth attendance, and full continuum of care completion compared with facilities that continued routine service delivery. These improvements were reinforced by implementation strategies informed by the CFIR ERIC framework, which targeted locally identified barriers related to leadership engagement, technical support, community involvement, and performance monitoring.

The findings provide real-world evidence that an integrated digital health and implementation package can strengthen continuity of maternal care within primary healthcare settings. Integrating eCHIS RMNCHN into routine workflows, supported by ongoing capacity building and supervision, may help sustain these gains. Further research is needed to assess long term outcomes, cost effectiveness, and the potential for expansion to other regions of the country.

## Materials and methods

### Ethics statement

Ethical approval for this study was obtained from the Institutional Review Board of the College of Health Sciences, Addis Ababa University (Protocol No. 059/24/SPH, issued on 01 November 2024). Permission to conduct the study was also obtained from the respective regional and facility-level health authorities prior to data collection.

Written informed consent was obtained from all participants before enrollment in the study. Participation was entirely voluntary, and participants were informed of their right to decline participation or withdraw from the study at any time without any consequences to the services they receive.

To ensure privacy and confidentiality, no personal identifiers were recorded on the data collection instruments. All collected data were stored in password-protected files accessible only to the research team, and were used solely for the purposes of this study.

### Study design and setting

A prospective controlled pre–post quasi-experimental study design was implemented in intervention and control PHCUs within East Shewa Zone of Eastern Ethiopia. The zone is located approximately 100 km southeast of Addis Ababa, covering an area of 10,232 km^2^ within the Great Rift Valley. It has an estimated population of 1,386,490, including 306,830 women of reproductive age. Administratively, the zone comprises 11 woredas, 2 town administrations, and 292 kebeles. Health services are delivered through 3 primary hospitals, 51 health centers, 5 comprehensive and 227 basic health posts [25]. The study was conducted in ten Primary Health Care Units (PHCUs), with five assigned to the intervention group and five to the control group. PHCUs were included in group if at least 85 percent of households and their members were registered in the electronic Community Health Information System (eCHIS), ensuring adequate digital infrastructure and reliable internet connectivity for the delivery of maternal health services through eCHIS

### Study population and eligibility criteria

The study population included pregnant women attending health centers in the intervention and control Primary Health Care Units (PHCUs). Women were eligible if they were at 26 weeks of gestation or earlier, had at least one previous birth, and resided permanently within the selected woreda and PHCU catchment areas. Enrollment was limited to women receiving their first antenatal care visit during the study recruitment period. Women who were critically ill, lived outside the designated catchment areas, or were temporary visitors to the participating health facilities were excluded.

### Sample size calculation and sampling procedure

The sample size was calculated using G*Power version 3.1.9.4 for the comparison of two independent proportions. The calculation assumed a two-sided alpha level of 0.05, 80% statistical power, and an equal allocation ratio between the intervention and control groups. The expected proportions were (P1 = 45.3%) and (P2 = 55.3%), based on the proportion of women who had four or more antenatal care visits. This represented an anticipated absolute difference of 10 percentage points in outcome of interest between the two groups [26]

Thus the minimum required sample size was 390 participants per group (a total of 780). After adjusting for a 10% non-response rate, the sample size increased to 434 per group, yielding a total of 868 participants. In practice, 418 women were enrolled in the intervention group and 421 in the control group, giving a total of 839 participants at baseline slightly less than the computed. At endline, outcome data were available for 409 women in each arm, which still exceeded the original requirement of 390 per group and thus maintained at least 80% statistical power to detect the pre-specified 10% point difference in the primary outcome.

The intervention group consisted of Primary Health Care Units (PHCUs) implementing the eCHIS RMNCHN module to enhance the provision of maternal health services across the continuum of care. Selection of intervention PHCUs was guided by predefined criteria, focusing on facilities located in woredas where more than 85% households and their members were registered in the eCHIS platform. This threshold served as an indicator of adequate digital infrastructure and readiness to deliver maternal and newborn health services through the eCHIS system. The control group included PHCUs that continued providing maternal health services through the conventional paper based or routine service delivery system. Control PHCUs were purposively matched to the intervention PHCUs based on similarities in catchment population size, the average monthly number of antenatal care attendees, and the number of skilled birth attendances. This matching approach was employed to ensure comparability between groups and to minimize baseline differences that could influence the outcome measures. To minimize the risk of information contamination, intervention and control PHCUs were purposively selected to be geographically distinct, located in opposite directions, and separated by an approximate distance of fifty kilometers.

Due to the limited number of eligible participants, every woman who met the inclusion criteria during the recruitment period was enrolled consecutively. This approach led to a census of all eligible arrivals within the thirty day enrollment window at each participating facility. Women were eligible if they were at 26 weeks of gestation or earlier, had at least one previous birth, and resided permanently within the selected woreda and PHCU catchment areas at the time of enrollment.

A total of 839 eligible pregnant women were consecutively enrolled during the baseline period, including 418 in the intervention PHCUs and 421 in the control PHCUs. Slight differences in baseline enrollment reflected natural variation in the number of eligible women presenting for first antenatal care visits during the recruitment window. After obtaining written informed consent, participant identifiers including full name, medical record number, ANC register serial number, kebele, gote, and phone number were recorded in a secure tracking sheet to facilitate follow up. The enrolled women were then prospectively followed for nine months. Outcome ascertainment relied primarily on review of antenatal, delivery, and postnatal care registers. When register information was incomplete or missing, data collectors conducted brief verification interviews in person or by phone. For women who could not be reached, additional tracing support was provided by Health Extension Workers and Women Development Groups, ensuring near complete outcome capture. At endline, 409 women were successfully followed in each study arm, corresponding to a low attrition rate of 2.85 percent in the control arm and 2.15 percent in the intervention arm. The participant enrollment and follow-up procedures are presented in Fig 3.

**Fig 3:**
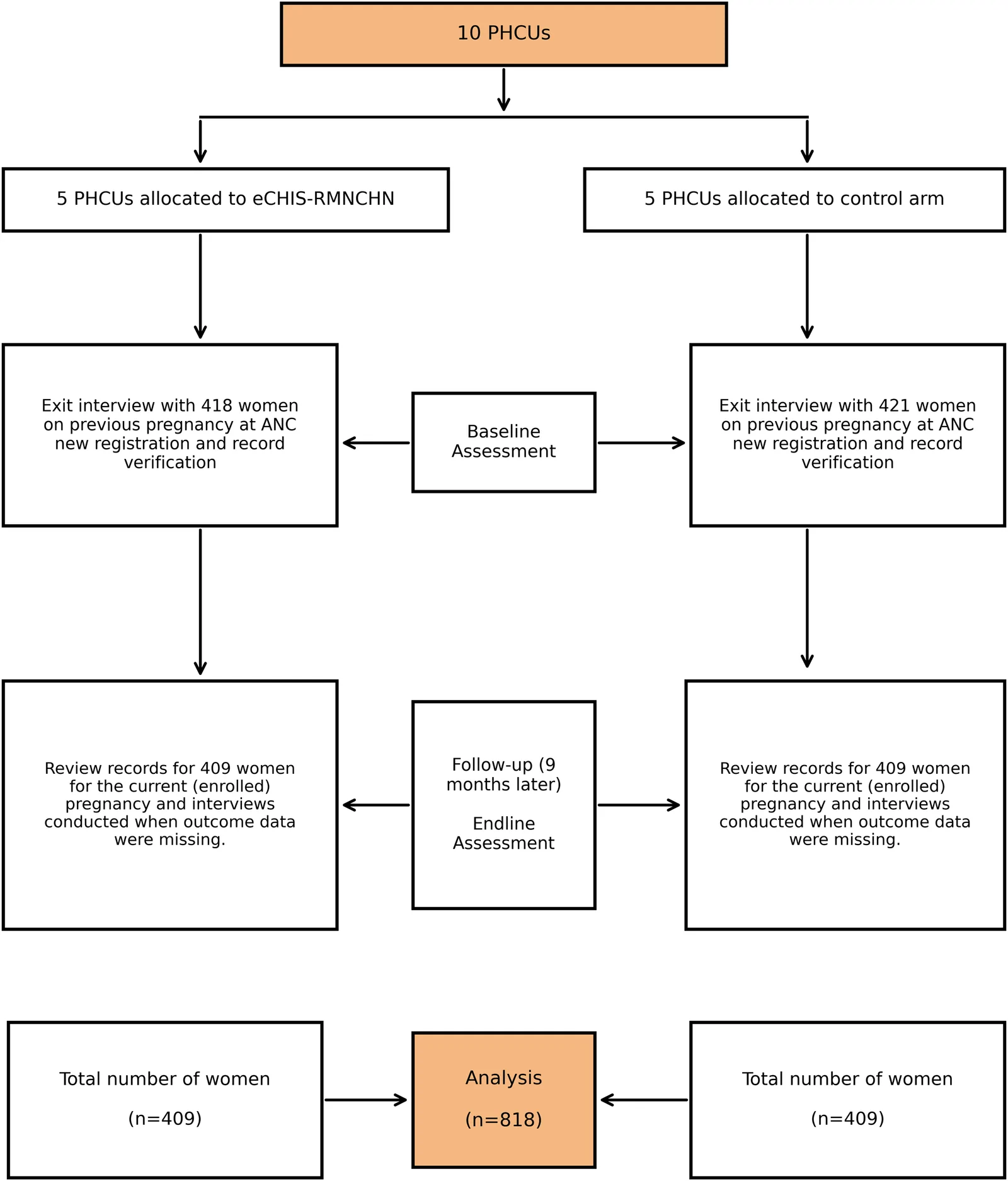
Schematic illustration of the sampling procedures used to select participants in the intervention and control groups, East Shewa Zone, 2025.

### Description of the Intervention

The intervention implemented the eCHIS-RMNCHN module to enhance the delivery and continuity of maternal health care. The Electronic Community Health Information System (eCHIS), introduced by the Ethiopian Ministry of Health in 2018, is a mobile based digital platform that supports service delivery at the community level. The Oromia Regional Health Bureau, in partnership with supporting stakeholders, implemented the eCHIS system in the East Shewa Zone. The RMNCHN module is a key component of this platform. It is designed to enhance maternal and newborn health by encouraging service uptake, improving the quality of care provided, and offering structured decision support to healthcare providers.

The module enables healthcare providers to assess maternal health status, identify danger signs, deliver targeted counseling, and maintain consistent client follow-up through automated appointment reminders. It also provides decision support that guides providers in determining whether services can be delivered at the primary level by health extension workers or if referral to higher-level care is warranted through the electronic referral system.

### Major components of the RMNCHN module

#### Counseling guides.

The module includes structured counseling guidelines that address obstetric danger signs and complications during pregnancy, childbirth, and the postpartum period. These guidelines improve the accuracy, consistency, and quality of counseling by outlining both the content and delivery approach required for effective client communication.

#### Decision support for healthcare providers.

This component serves as a clinical decision support tool, enabling providers to systematically evaluate the health status of pregnant women and identify conditions that require higher-level care. The module incorporates standardized assessment questions and generates recommendations based on client responses, helping providers determine whether routine maternal and child health services are appropriate or if referral to more specialized professionals is necessary.

#### Electronic referral system.

The module supports an integrated electronic referral mechanism between health extension workers and midwives, ensuring timely communication, prompt case management, and continuous follow-up across different levels of care.

#### Appointment reminders and visit prioritization.

The application automatically generates appointment dates based on the gestational age of pregnant women and displays upcoming visits using a four color coded reminder system, as shown in the figure. This visual mechanism enables healthcare providers to quickly assess the urgency of each scheduled visit. Appointments that are three or more days in the future appear in gray, indicating that the visit is forthcoming. Visits that fall within two days before or after the due date are shown in green, signifying that the appointment is on time. When a client is three to six days late from the scheduled date, the system displays the visit in yellow to indicate that the appointment has been missed and requires follow up. Critically overdue visits, which are seven or more days late, are highlighted in red to signal the need for immediate attention. This color coded system allows providers to rapidly identify visit status and ensure timely and proactive maternal health care.

#### Reporting functionality.

Aligned with the revised HMIS indicators, the module incorporate maternal and child health service indicators to facilitate streamlined report generation and seamless integration into DHIS2. This functionality reduces the reporting burden on providers, improves efficiency, and enhances the timeliness of routine reports.

### Implementation strategies for the eCHIS-RMNCHN module

Using the CFIR–ERIC implementation science framework, core strategies were developed and executed to strengthen adoption, optimize functionality, and enhance use of the eCHIS-RMNCHN module [10]. These implementation strategies were not arbitrary; they were developed based on a prior assessment of facilitators and barriers to eCHIS implementation conducted in the same study setting. That assessment used the CFIR–ERIC framework to identify key determinants and map them to appropriate implementation strategies. The strategies applied in the present study were therefore pre-planned, context-specific, and grounded in established implementation-science methods. All strategies were implemented during the follow-up period in collaboration with Regional health bureau, zonal and woreda health offices, PHCUs, and community structures.

1. **Strengthen Leadership Engagement and Advocacy**
2. Targeted engagement and advocacy activities were conducted at zonal, woreda, and kebele levels to address limited leadership attention toward eCHIS. Leadership orientation sessions were organized for health office heads, PHCU directors, and kebele administrators, emphasizing the strategic importance of eCHIS and its role in improving maternal and newborn health outcomes. These sessions enhanced leaders’ understanding of their responsibilities in supervision, prioritization, and resource mobilization. As a result, leaders demonstrated stronger commitment through active participation in review meetings, closer monitoring of performance, and endorsement of eCHIS-related activities within their administrative units.
3. **Enhance Technical Support and Training Programs**
4. Continuous technical support was provided to address gaps in post-training follow-up and challenges related to system functionality. Health Information Technicians (HITs) conducted routine mentorship visits to PHCUs, offering hands-on coaching, addressing device-related issues, and resolving problems such as connectivity failures, data synchronization delays, and interruptions in electronic referrals. Refresher training was delivered to health extension workers and midwives to reinforce system skills and ensure accurate and timely data entry. These efforts improved user competency, reduced documentation interruptions, and increased overall confidence in operating the RMNCHN module.
5. **Foster Community-Level Engagement and Ownership**
6. Community structures were mobilized to increase awareness and support for eCHIS, addressing the low community engagement identified in the assessment. Women Development Groups, community volunteers, and local leaders were oriented on the purpose of eCHIS and their roles in maternal health tracking. These activities expanded community involvement in identifying pregnant women, following up missed appointments, and encouraging timely facility visits. Improved community participation created a supportive environment that strengthened maternal service uptake and reinforced the value of the digital system at the local level.
7. **Establish a Robust Feedback and Performance Monitoring System**
8. Structured feedback mechanisms were introduced to support performance improvement and strengthen accountability. Routine review meetings incorporated eCHIS dashboard data to monitor service delivery progress, identify gaps, and agree on corrective actions. Health workers received regular updates on indicators such as ANC follow-up, skilled birth attendance, referral completion, and postpartum service coverage. These feedback loops improved clarity on expectations, enhanced motivation, and supported continuous quality improvement across PHCUs.
9. **Integrate eCHIS into Health Worker Incentive Programs**
10. To address demotivation associated with limited recognition, non-financial incentives were integrated into routine management practices. High-performing users of the RMNCHN module were acknowledged during review meetings, and certificates of recognition were issued to reinforce positive practices. This approach improved morale, encouraged consistent and accurate data entry, and fostered a stronger sense of ownership and commitment among health workers. The recognition contributed to improved system uptake and greater long-term sustainability of eCHIS use**.**

### Data collection instruments, procedures, and quality assurance

Data collection was conducted in two phases using a structured questionnaire developed in English and translated into Amharic and Afan Oromo. The tool was reviewed by subject matter experts, refined based on their feedback, and pretested in facilities not included in the study. Data collectors and supervisors received comprehensive training on interviewing techniques, register abstraction, and ethical procedures prior to fieldwork.

Baseline data collection for the effectiveness study was conducted from January 5 to February 5, 2025. Ten trained data collectors, each assigned to one health facility, carried out the data collection with support from two supervisors responsible for ongoing quality assurance. Supervisors performed spot checks, back-checked interviews, and verified information abstracted from facility registers. Facility staff assisted only in locating the appropriate registers. All data were collected electronically using the KoboToolbox platform.

At baseline, eligible pregnant women attending their first antenatal care visit were interviewed face to face. Information on socio-demographic characteristics, reproductive history, and details of the most recent pregnancy was collected and cross-checked with antenatal, delivery, and postnatal registers.

Following nine months of follow-up during which the eCHIS RMNCHN module and CFIR–ERIC informed implementation strategies were actively deployed, endline outcome data were collected from October 5 to November 5, 2025. Outcome information was extracted from antenatal, delivery, and postnatal care registers, including completion of four or more antenatal visits, skilled birth attendance, and receipt of postnatal care within forty-eight hours. When records were incomplete, verification interviews were conducted in person or by telephone. Supervisors reviewed a sample of register abstractions and verification interviews to ensure accuracy and consistency.

### Outcome

The primary outcome was completion of the continuum of care (CoC), defined as receipt of all three essential maternal health services: (i) four or more antenatal care visits (ANC4+), (ii) skilled birth attendance (SBA), and (iii) postnatal care (PNC) within 48 hours of childbirth. For analytical purposes, stage-specific indicators were also constructed and coded as binary variables [27].

Completion of care at the pregnancy stage assessed whether a woman achieved ANC4+ and was coded as 1 for those who received four or more antenatal visits and 0 otherwise. Completion of care at the delivery stage examined attainment of both ANC4+ and SBA, coded as 1 for women who met both criteria and 0 for those who achieved ANC4+ but did not receive SBA. Completion of the full continuum at the postpartum stage represented receipt of ANC4 + , SBA, and PNC within 48 hours after delivery, coded as 1 for women who received all three services and 0 otherwise.

### Data processing and analysis

Data were cleaned, coded, and analyzed using STATA version 17. Descriptive statistics were computed to summarize participants’ baseline and endline characteristics as well as key study variables. To assess pre-intervention comparability, descriptive maternal health service indicators were compared between the intervention and control PHCUs before implementation. Differences in proportions were assessed using Pearson chi-square tests, and absolute percentage-point differences with 95% confidence intervals were reported. The primary impact analysis employed a Prospective controlled pre–post Difference-in-Differences framework using a woman-specific fixed-effects regression model. Each woman contributed two observations: one corresponding to her most recent previous pregnancy (p = 0) and the other corresponding to the prospectively followed index pregnancy (p = 1). The primary analytical equation was defined as follows:


$$\begin{array}{c} Yip=\alpha i+\beta 1Postp+\beta 2(Interventioni\times Postp)+\varepsilon ip, \end{array}$$

Where:

- Yip represents completion of the maternal continuum of care for woman i during pregnancy episode p.
- αi denotes the woman-specific fixed effect and accounts for characteristics that remain unchanged across the two pregnancy episodes.
- β1 symbolizes the average change in maternal continuum-of-care completion from the previous pregnancy to the index pregnancy in the control group.
- Postp is the pregnancy-period indicator, coded 0 for the previous pregnancy and 1 for the index pregnancy.
- β2 represents the controlled pre–post Difference-in-Differences estimate, measuring the additional change in the intervention group relative to the control group.
- Interventioni × Postp is the interaction term identifying women in the intervention group during the index-pregnancy period.
- εip is the error term, representing unexplained variation in maternal continuum-of-care completion.

Statistical significance was assessed using a two-sided alpha level of 0.05. The primary parameter of interest was β2, the coefficient of the interaction term (Intervention_i_ × Postp). This coefficient represents the controlled pre–post Difference-in-Differences estimate and quantifies the additional change in maternal continuum-of-care completion among women in the intervention group compared to the corresponding change among women in the control group.

Effect estimates are presented with 95% confidence intervals and corresponding p-values. A p-value of less than 0.05 was considered statistically significant, and confidence intervals that did not include zero were interpreted as evidence of a statistically meaningful intervention effect on the outcome. To account for potential intra-facility correlation, cluster-robust standard errors were estimated at the health facility level. In addition, wild cluster bootstrap procedures were applied to assess the robustness of the findings in light of the limited number of clusters.

To assess the robustness of the estimated intervention effects to observed baseline imbalances, sensitivity analyses were conducted by extending the woman-specific fixed-effects Difference-in-Differences models to include interactions between the post-intervention indicator and all baseline characteristics that differed significantly between the intervention and comparison groups. These included age group, religion, ethnicity, parity, history of poor fetal outcome, and other variables with statistically significant baseline imbalances. As these characteristics were treated as time-invariant, their main effects were absorbed by the woman-specific fixed effects. Their interactions with the post-intervention indicator were therefore included to allow temporal changes in the outcomes to vary across baseline subgroups. These analyses assessed whether the estimated intervention effects were sensitive to differential outcome changes associated with measured baseline characteristics. However, they could not eliminate confounding from unmeasured time-varying contextual or health-system factors.

The Difference in Differences approach relies on the parallel trends assumption: in the absence of the intervention, changes in outcomes over time would have been similar between the intervention and control groups. Because only one pre-intervention measurement was available, this assumption could not be empirically tested. However, the two groups exhibited comparable baseline levels for the primary outcome, and evidence from similar settings suggests broadly similar secular trends. These factors support, but cannot fully verify, the plausibility of the parallel trends assumption.

## Supporting information

S1 Data Minimal dataset. (DTA)

## Abbreviations

ANC: Antenatal Care
ANC4+: Four or More Antenatal Care Visits
AOR: Adjusted Odds Ratio
CFIR: Consolidated Framework for Implementation Research
CI: Confidence Interval
CoC: , Continuum of Care;
DiD: Difference-in-Differences
eCHIS: , Electronic Community Health Information System;
ECD: Early Childhood Development
ERIC: Expert Recommendations for Implementing Change
HEWs: , Health Extension Workers;
HEP: Health Extension Program;
HITs: , Health Information Technicians;
HMIS: Health Management Information System
LMIC: Low- and Middle-Income Countries
MCH: Maternal and Child Health;
MMEIG: Maternal Mortality Estimation Inter-Agency Group
MoH: Ministry of Health
PHCU: Primary Health Care Unit
PNC: Postnatal Care;
SBA: Skilled Birth Attendance;
SDG: Sustainable Development Goal
UNICEF: United Nations Children’s Fund
WHO: World Health Organization
